# Development and Verification of Glutamatergic Synapse-Associated Prognosis Signature for Lower-Grade Gliomas

**DOI:** 10.3389/fnmol.2021.720899

**Published:** 2021-10-28

**Authors:** Liguo Ye, Yang Xu, Ping Hu, Long Wang, Ji’an Yang, Fan’en Yuan, Yixuan Wang, Chunyu Zhang, Daofeng Tian, Qianxue Chen

**Affiliations:** Department of Neurosurgery, Renmin Hospital of Wuhan University, Wuhan, China

**Keywords:** lower-grade glioma, glutamatergic synapses, risk signature, tumor immune microenvironment, immunity profile

## Abstract

**Background:** Lower-grade glioma (LGG) is the most common histology identified in gliomas, a heterogeneous tumor that may develop into high-grade malignant glioma that seriously shortens patient survival time. Recent studies reported that glutamatergic synapses might play an essential role in the progress of gliomas. However, the role of glutamatergic synapse-related biomarkers in LGG has not been systemically researched yet.

**Methods:** The mRNA expression data of glioma and normal brain tissue were obtained from The Cancer Genome Atlas database and Genotype-Tissue Expression, respectively, and the Chinese Glioma Genome Atlas database was used as a validation set. Difference analysis was performed to evaluate the expression pattern of glutamatergic synapse-related genes (GSRGs) in LGG. The least absolute shrinkage and selection operator (LASSO) Cox regression was applied to construct the glutamatergic synapse-related risk signature (GSRS), and the risk score of each LGG sample was calculated based on the coefficients and expression value of selected GSRGs. Univariate and multivariate Cox regression analyses were used to investigate the prognostic value of risk score. Immunity profile and single-sample gene set enrichment analysis (ssGSEA) were performed to explore the association between risk score and the characters of tumor microenvironment in LGG. Gene set variation analysis (GSVA) was performed to investigate the potential pathways related to GSRS. The HPA database and real-time PCR were used to identify the expression of hub genes identified in GSRS.

**Results:** A total of 22 genes of 39 GSRGs were found differentially expressed among normal and LGG samples. Through the LASSO algorithm, 14-genes GSRS constructed were associated with the prognosis and clinicopathological features of patients with LGG. Furthermore, the risk score level was significantly positively correlated with the infiltrating level of immunosuppressive cells, including M2 macrophages and regulatory T cells. GSVA identified a series of cancer-related pathways related to GSRS, such as P13K-AKT and P53 pathways. Moreover, ATAD1, NLGN2, OXTR, and TNR, hub genes identified in GSRS, were considered as potential prognostic biomarkers in LGG.

**Conclusion:** A 14-genes GSRS was constructed and verified in this study. We provided a novel insight into the role of GSRS in LGG through a series of bioinformatics methods.

## Introduction

Glioma is the most frequent primary malignant brain tumor, with about 10,000 new cases reported every year (Lapointe et al., 2018). Indeed, some brain lower-grade glioma (LGG), including the WHO II, III grade, could remain indolent for years, while others rapidly progress to glioblastoma. Therefore, the survival of patients with LGG ranges from 15 years to 1 year (Zhao et al., 2019). At present, the available primary treatment for LGG is still surgical resection (Han et al., 2017). However, due to the silent clinical characteristics of LGG, most patients miss the suitable opportunity for surgery. Besides, the combination of radiotherapy and temozolomide chemotherapy, the first-line adjuvant strategy, still has a high risk of acquired primary resistance (Olubajo et al., 2020). It is partly a result of a poor understanding of the exact etiology and pathogenesis for gliomas. Therefore, novel effective biomarkers of LGG patients were vital to be identified for applying to therapy strategies.

Despite the process of understanding the molecular pathogenesis of glioma, the prognosis of patients and treatment effect of this tumor remained poor (Chen et al., 2015). In malignant glioma tissues interacting with neurons, glioma cells foster a tumor-favorable microenvironment to promote self-proliferation and escape from the immune response (Guo et al., 2019). Recent studies have shown that gliomas could manipulate normal elements of neuronal plasticity and development in the tumor microenvironment (TME), which could create an abnormal connection between neurons and tumor cells by neuronal glioma synapses (NGS; Venkatesh et al., 2015, 2019; Venkataramani et al., 2019). Meanwhile, the electrical activity of neurons mediates the depolarization of the calcium signal network of glioma cells through NGS, thus increasing the proliferation and invasion of the tumor, leading to the progression of glioma (Luk and Sadikot, 2004; Venkataramani et al., 2019; Venkatesh et al., 2019; Lim-Fat and Wen, 2020). The glutamatergic synapses are considered the main functional structures of NGS (Luk and Sadikot, 2004; Venkataramani et al., 2019; Lim-Fat and Wen, 2020). The glutamatergic synapse may play a crucial role in mediating neuronal-glial circuits in TME; it involves three vital elements: presynaptic neurons, postsynaptic neurons, and astrocytes (Findley et al., 2019). Venkataramani et al. (2019) further detected the biological markers of postsynaptic structure, including HOMER1, HOMER2, HOMER3, and glutamatergic vesicles in microtubules connecting glioma cells with normal neurons and glioma cells. In addition, neuroligin-3 (NLGN3) has been considered to be involved in the phosphoinositide 3-kinase/mammalian target of rapamycin (PI3K-mTOR) signaling pathway to promote the proliferation of gliomas (Venkatesh et al., 2015). It was reported that NLGN3 is highly expressed in the co-culture environment of neurons and glioma. After inhibiting the expression of NLGN3, the growth of glioma cells was significantly suppressed. Furthermore, in the medium with high expression of NLGN3, the density of synaptic connections between gliomas and neurons was significantly higher than that in the culture environment with the inhibited expression of NLGN3. It suggested that NLGN3, as a critical synaptic adhesion factor, may directly mediate the invasion and proliferation of glioma by participating in the synaptic formation mechanism (Venkatesh et al., 2017). Therefore, glutamatergic synapses might mediate the profession and progression of gliomas in the brain.

The tumor immune microenvironment (TIME) components could direct response to related treatment and be a vital determinant of tumor-immune interactions (Grabovska et al., 2020). Moreover, tumor-infiltrating immune cells (TIICs) have proven beneficial effects for tumor progression (Lai et al., 2019). Immunotherapy is undergoing rapid advances and has become a novel treatment strategy in various cancers (Lu et al., 2020; Waaler et al., 2020). Mainly, immune checkpoint blockade (ICB) has emerged as the most promising immunotherapy modality in cancer (Wei et al., 2018). However, only a small number of patients were sensitive to the ICB treatment (Sharma et al., 2017). Therefore, it would benefit from avoiding immune-related adverse events and decreasing treatment costs by identifying novel biomarkers combined with immune checkpoints (ICPs). The involvement of microglia, the CNS resident immune cells, and phagocytes in synaptic refinement has been widely established (Scott-Hewitt et al., 2020). Moreover, the immune synapse connections among immune cells, such as T-cells and antigen-presenting cells, are essential means of intracellular communication in immune cells in coordinating many functions (Davis and Dustin, 2004). Consequently, it is of great significance to explore the influence of glutamatergic synapse-related genes (GSRGs) in TIME and identify glutamatergic synapse-related biomarkers in LGG, which may be practical for understanding the underlying pathogenesis of gliomas.

Here, we conjectured that the interaction between glutamatergic synapses and the immune status has a particular significance for the prognosis of glioma. A novel glutamatergic synapse-related risk signature (GSRS), developed and verified through various bioinformatics methods, might be incorporated into the existing clinicopathological characteristics and staging system to improve the prognosis of LGG patients. In addition, the association between the risk signature and immune profiles will be investigated in LGG.

## Data and Methods

### Public Data and Samples Collection

We collected whole-genome RNA-seq expression data and clinical and molecular information and eliminated incomplete clinical information along with lacking prognostic information from 529 LGG (including WHO II-III grade glioma) samples in The Cancer Genome Atlas (TCGA) database^1^. A total of 625 LGG (WHO II-III grade) glioma samples were screened out from Chinese Glioma Genome Atlas (CGGA)^2^ part A and B, which was merged, standardized, and then used as a validation set. In this study, cases with ≤30 days of survival or those with no survival data were eliminated since they might die of fetal complications (including hemorrhage, intracranial infection, and heart failure) rather than LGG. Among the mentioned cases, 447 LGG samples in TCGA and 592 LGG samples in CGGA with complete mRNA expression data and corresponding clinical materials were selected for subsequent analyses. In addition, 940 normal brain samples (including brain tissues in different parts such as cortex, cerebellum, and brainstem) with complete mRNA_seq data were used as a control set. In addition, considering that batch effects may exist between or within different databases, we used the “normalizeBetweenArrays” function (Bolstad et al., 2003; Ritchie et al., 2015) of R package “limma” to remove multiple batch effects merging the mRNA_seq data of TCGA and GTEx, and CGGA part A and B.

### Patient Samples

This study was approved by the Institutional Ethics Committee of the Faculty of Medicine at Renmin Hospital of Wuhan University. Informed consent was obtained from all patients whose tissues were used. In total, six control samples from patients with cerebral hemorrhage and 24 lower-grade glioma samples (WHO grade II-III, 10 and 14 samples) were collected during May 2019 and April 2021. All patients were not treated with chemotherapy or radiotherapy before surgery. The independent samples from our hospital will be used to verify the mRNA expression level of hub genes of GSRS in LGG.

### Obtaining Glutamatergic Synapse-Related Gene Sets

Gene sets, ‘‘GOBP_NEGATIVE_REGULATION_OF_SYNAPT IC_TRANSMISSION_GLUTAMATERGIC’’ and ‘‘GOBP_POSI TIVE_REGULATION_OF_SYNAPTIC_TRANSMISSION_GLU TAMATERGIC,’’ were obtained from the Molecular Signatures Database^3^.

### Identify Differentially Expressed Genes Between Lower-Grade Glioma and Normal Tissues

Genotype-Tissue Expression and TCGA-LGG databases were combined as a training set. Though R package “limma” (Ritchie et al., 2015), differentially expressed genes (DEGs) were identified from GSRGs; false discovery rate (FDR) less than 0.05 and abs of logFC larger than 1 were set as the criterion.

### Protein–Protein Interaction Network Analysis

The protein--protein interaction (PPI) network of 39 GSRGs was constructed in the STRING database^4^. Nodes with confidences of interactive relationships larger than 0.4 were shown.

### Genomic Alterations of 39 Glutamatergic Synapse-Related Genes

Copy number variation (CNV) amplification, CNV deep deletion, in-frame mutation, truncating mutation, missense mutation, and fusions of these 39 genes were analyzed in the cBioPortal dataset^5^.

### Construction of Glutamatergic Synapse-Related Risk Signature

Univariate Cox regression was performed through the “survival” R package to assess the prognostic value of the GSRGs in LGG (genes with *p*-value < 0.05 were selected for further study). Based on the survival time, survival status, and expression level of prognosis-related genes of patients with LGG, the least absolute shrinkage and selection operator (LASSO) regression algorithm (Miller, 2009) was used to formulate a risk signature (the penalty parameter λ was chosen based on 10-fold cross-validation). Genes and their regression coefficients were then obtained according to the most suitable λ value.

The formula was given as follows:


Riskscore=exprgene(1)×coefficientgene(1)+exprgene(2)×

coefficientgene⁢(2)+⋯+exprgene⁢(n)×coefficientgene⁢(n)

where *n* is the number of prognostic genes, exprgene is the expression value of the gene, and coefficientgene is the coefficient of the gene in the risk signature.

### Principal Components Analysis

According to the median value of risk score calculated, LGG samples were assigned into high- and low-risk groups, and the principal component analysis (PCA) was used to confirm the differences between the groups through dimensionality reduction of the mRNA expression data in both TCGA-LGG and CGGA.

### Prognostic Analysis of Glutamatergic Synapse-Related Risk Signature

The prognostic significance of the GSRS in LGG was evaluated by Kaplan–Meier survival curves and Cox regression analysis in the training and verification datasets (log-rank test *p*-value < 0.05 was considered significant). In addition, the receiver operating characteristic (ROC) curves of GSRS and other clinical risk factors for predicting 1-, 3-, and 5-year overall survival (OS) of patients with LGG were performed, and the area under the ROC curves (AUCs) was calculated. Swets (1988) established three categories to determine the accuracy of a diagnostic technique based on the AUC-ROC: high accuracy (0.9 < AUC-ROC ≤ 1), moderate accuracy (0.7 < AUC-ROC ≤ 0.9), and finally, low accuracy (0.5 < AUC-ROC ≤ 0.7).

### Clinicopathological Relevance of the Glutamatergic Synapse-Related Risk Signature

Patients were separated into high- and low-risk groups in both training and verification cohorts. The chi-square test performed the difference analysis of risk score among clinicopathological characteristics, including WHO grade, IDH mutation status, 1p19q co-deletion status, age group, and gender. *p*-value < 0.05 was considered significant.

### Tumor-Infiltrating Immune Cells Profiles

The abundance profile of immune cells was estimated by the CIBERSORT computational method in the low- and high-risk groups separately. Pearson correlation analysis and the Wilcoxon test investigated the correlation between the fraction of TIICs and risk score in both TCGA-LGG and CGGA cohorts (*p* < 0.05 was considered significant). In addition, based on the ESTIMATE algorithm, the immune score, stromal score, and tumor purity of each LGG sample were calculated with the “estimate” package (Ciardullo et al., 2020).

### Single-Sample Gene Sets Enrichment Analysis

The vital genes of 29 immune-related pathways were extracted from the study of Bindea et al. (2013). Single-sample GSEA (ssGSEA; Finotello and Trajanoski, 2018) was used to calculate the level of tumor-infiltrating immune cells based on melanoma mRNA TPM data. In addition, the difference analysis of enrichment degree of gene hallmarks with 29 kinds of immune-related hallmarks was performed among low- and high-risk groups in the training and verification sets (*p*-value < 0.05 was considered significant).

Moreover, considering the importance of ICPs and immunogenic cell death (ICD) modulators in cancer immunity, we then analyzed their expression levels among low- and high-risk groups.

### Mutational Status Analysis

In the TCGA-LGG dataset, tumor somatic mutational load was calculated as a total number of mutations identified in each sample. In addition, the prognostic value of tumor mutation burden (TMB) in LGG was explored, and the mutational status was calculated and compared in low- and high-risk groups by R package “maftools.”

### Gene Set Variation Analysis

Hallmark gene sets, which summarize and represent specific, well-defined biological states or processes and display coherent expression, were downloaded from the Molecular Signatures Database and chosen for further analysis. GSVA of hallmark gene sets was implemented among low- and high-risk groups in TCGA-LGG using the R package “GSVA” (Hänzelmann et al., 2013).

### Verification of Hub Genes of Glutamatergic Synapse-Related Risk Signature

The prognosis-related genes of GSRS were identified through K--M survival curves in both training and validation sets. In addition, according to The Human Protein Atlas (HPA) database^6^, the protein level of genes identified among normal brain and LGG tissues was investigated.

### RNA Extraction and Quantitative Real-Time PCR

The extraction of RNA of prognosis-related genes from tissues and cells was carried out by TRIzol reagent (Invitrogen, Carlsbad, CA, United States). The PrimeScript RT Reagent Kit (RR047A, TaKaRa, Japan) was used to synthesize cDNA. We used SYBR Premix Ex Taq II (RR820A, TaKaRa, Kusatsu, Japan) and Bio-Rad CFX Manager 2.1 real-time PCR Systems (Bio-Rad, Hercules, CA, United States) to detect mRNA levels following the specifications provided by the manufacturers. The relative Ct method was adopted to compare the data of the experimental group and the control group, and GAPDH was set as an internal control.

## Results

### Genetic Alterations of Glutamatergic Synapse-Related Genes in Lower-Grade Glioma

**FIGURE 1:**
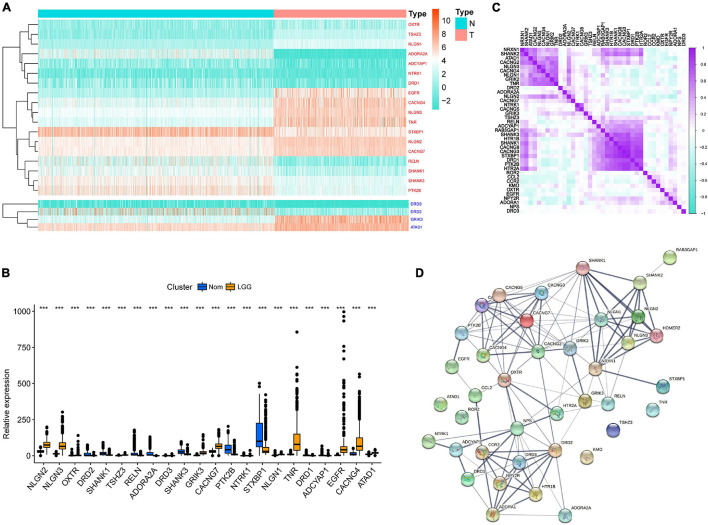
The genomic characterization of glutamatergic synapse-related genes (GSRGs). **(A)** Heatmap for differentially expressed GSRGs; genes with red color are involved in positive regulation of glutamatergic synaptic transmission, while genes with blue color mainly participate in negative regulation of glutamatergic synaptic transmission. **(B)** Boxplot for differentially expressed GSRGs. **(C)** Correlation plot for GSRGs; purple squares indicate positive correlation and turquoise squares indicate inverse correlation. **(D)** Protein-protein interaction network of GSRGs in STRING database. ***p < 0.001.

Through difference analysis of 39 GSRGs in the training set, 22 genes were found differentially expressed among normal and LGG samples, as shown in Figures 1A,B. Upregulated and downregulated GSRGs and corresponding logFC values were arranged in Supplementary Table 1. Subsequently, through Spearman’s correlation analysis, we found a strong expression correlation among GSRGs (Figure 1C). Furthermore, the PPI network analysis confirmed a strong co-expression correlation among the GSRGs (Figure 1D). In addition, to better understand the genomic characteristics of GSRGs in LGG, the mutation analysis performed in the cBioPortal database showed the copy number variation and somatic mutational status of GSRGs (Supplementary Figure 1).

### Construction and Verification of Glutamatergic Synapse-Related Risk Signature

**FIGURE 2:**
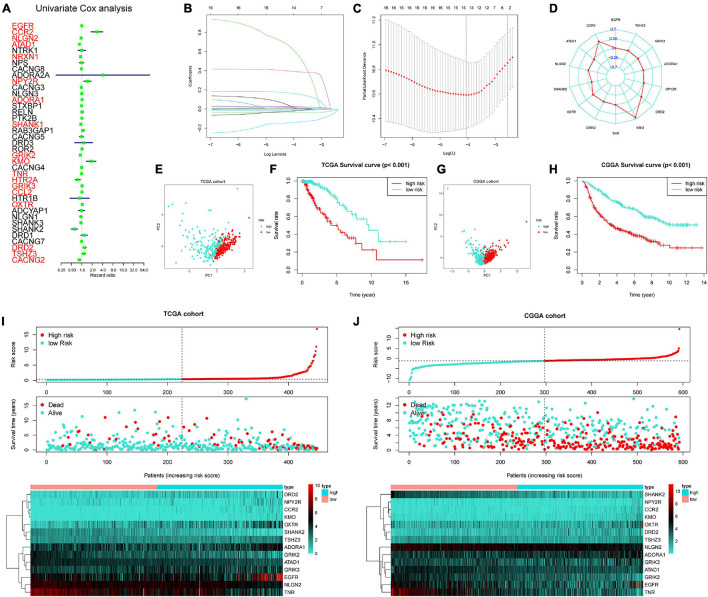
Construction of 14-genes glutamatergic synapse-related risk signature (GSRS). **(A)** Forest plot for the survival analysis of LGG patients using a univariate Cox model after adjustment for GSRGs; red color represents *p* < 0.05. **(B)** The craft plot for partial likelihood deviance in LASSO, different colors represent different genes in GSRS. **(C)** Partial likelihood deviance as a function of regularization parameter λ in the training dataset. Each red point marks a λ value along regularization paths, and gray error bars represent confidence intervals for the cross-validated error rate. The left vertical dotted line marks the minimum error, whereas the right vertical dotted line marks the most significant λ value, the error of which is within 1 SD of the minimum. The horizontal row of numbers above the plot marks the gene number in each condition upon shrinkage and selection based on linear regression. **(D)** Radar diagram of efficiency of the 14 genes in GSRS; the closer the red dot is to the outside, the greater the value it represents. **(E)** Principal component analysis (PCA) of LGG samples in TCGA; dots in turquoise represent samples in high-risk groups and dots in red represent samples in low-risk groups. **(F)** Overall survival analysis of risk score for LGG patients in TCGA. **(G)** PCA in CGGA-LGG. **(H)** Survival analysis in CGGA-LGG. According to training **(I)** and validation **(J)** sets, the distribution of risk score, corresponding OS, and gene expression are listed in the picture from top to bottom.

A total of 18 prognosis-related genes (*p* < 0.05) were identified from 39 GSRGs through univariate Cox analysis for further LASSO regression analysis (Figure 2A). After validation, using the LASSO with fixed λ = 10-5, the optimal model included 14 genes as features, including EGFR, CCR2, ATAD1, NLGN2, SHANK2, OXTR, GRIK2, TNR, KMO, DRD2, NPY2R, ADORA1, GRIK3, and TSHZ3. These 14 genes and their corresponding coefficients were sorted out in Supplementary Table 2. Then, the risk score of each patient was calculated according to the mRNA expression value of each risk gene and the corresponding coefficients (Figures 2B–D). As visualized in PCA, low- and high-risk clusters could well be distinguished through the median value of GSRS. In addition, survival analysis performed in training and validation sets also showed distinct clinical outcomes between low- and high-risk groups (Figures 2E–H). Moreover, the distributions of risk gene expression, risk score, and survival status were plotted in the GSRS of TCGA and CGGA-LGG cohorts (Figures 2I,J). All these results suggested that the risk score based on GSRS could be a better indicator for predicting the prognosis of patients with LGG than other clinical factors.

### The Risk Score Could Be an Independent Factor to Predict the Overall Survival of Lower-Grade Glioma Patients

**FIGURE 3:**
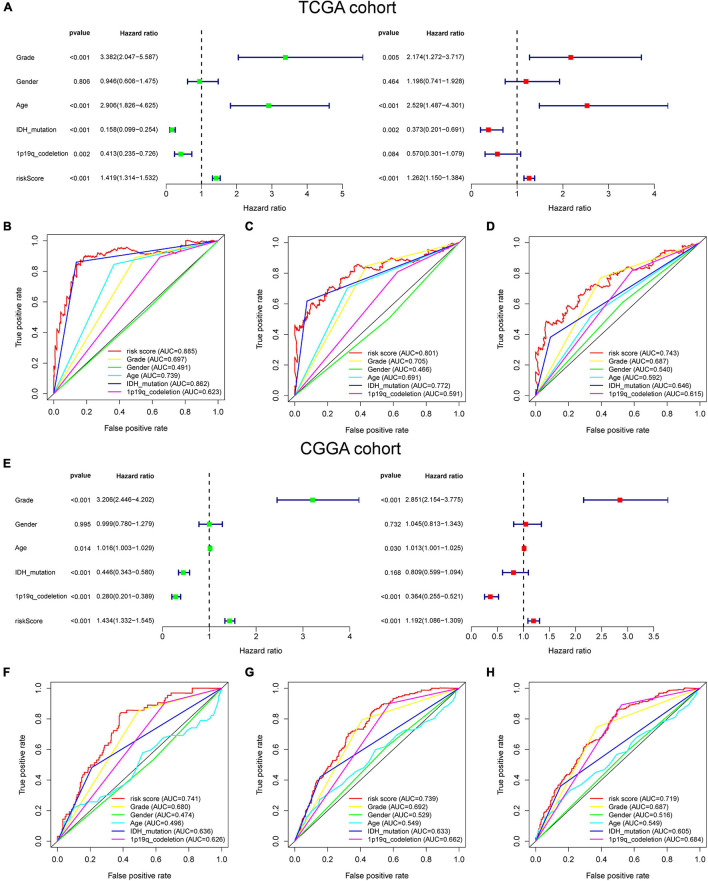
The prognostic value of GSRS. **(A)** In the training set, forest plot on the left for the univariate Cox test evaluating the association of the risk score and clinical factors with patient OS, and forest plot on the right for the multivariate Cox analysis identifying independent risk factors for the OS of patients. The ROC curve of risk score and clinical factors for predicting 1- **(B)**, 3- **(C)**, and 5-year **(D)** OS. **(E)** In the validation set, univariate and multivariate COX analysis of risk score and clinical factors. ROC curve of risk score compared with other clinical factors for predicting 1- **(F)**, 3- **(G)**, and 5-year **(H)** OS.

For better exploring the significance of our GSRS in predicting the prognosis of patients independently, univariate combining with multivariate COX analysis was conducted (Figure 3A), and we found that the risk score might serve as an independent factor for predicting the OS of patients for the TCGA-LGG cohort (hazard ratio HR: 1.262, *p* < 0.001), and the risk score achieved a higher area under the AUC-ROC compared with all other prognosis-related clinical factors, including WHO grade, age, IDH mutational status, 1p19q co-deletion status, and gender. The AUC of a risk score for 1-, 3-, and 5-year OS of patients in the training set was 0.885, 0.801, and 0.743, respectively (Figures 3B–D). These results were validated in the CGGA-LGG cohort, and HR of the risk score in multivariate COX regression was 1.192 (Figure 3E, *p* < 0.001), and the AUC of a risk score for 1-, 3-, and 5-year OS of patients in CGGA-LGG were 0.741, 0.739, and 0.719, respectively (Figures 3F–H).

### Relationship Between Glutamatergic Synapse-Related Risk Signature and the Clinicopathological Features

**FIGURE 4:**
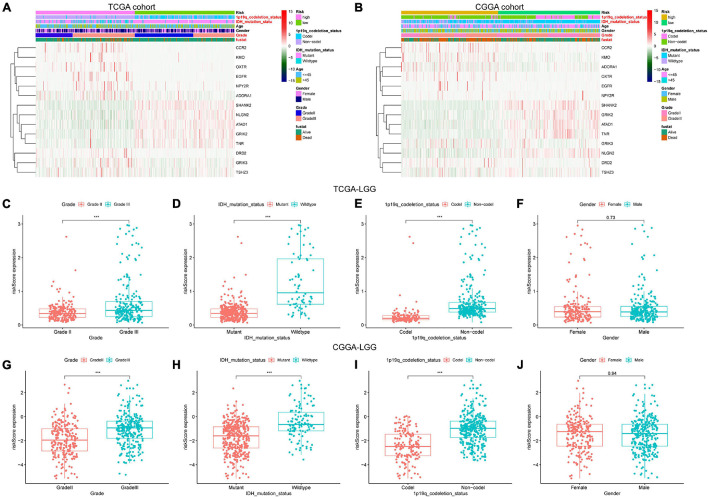
The association between risk score and clinicopathological factors. Heatmap of the correlations between risk score and clinicopathological characteristics of LGG in TCGA **(A)** and CGGA **(B)** cohorts; factors with red color are significantly correlated with the risk score. Distribution of glutamatergic synapses-related risk signature among LGG patients stratified by WHO grade, IDH status, 1p/19q co-deletion status, and gender in TCGA **(C–F)** and CGGA **(G–J)** cohorts. ***p < 0.001.

We screened out 447 and 592 cases with sufficient data on age, gender, WHO grade, IDH mutational status, and 1p19q co-deletion status in training and validation sets, respectively. Chi-square tests were performed for the comparisons of the distribution of clinical factors among different risk groups with the R function “chisq.test.” The results of the chi-square tests in TCGA and CGGA cohorts were presented in Table 1. We found that the risk scores obtained based on GSRS showed a significant correlation with WHO grade, IDH mutational status, and 1p19q co-deletion status in TCGA and CGGA cohorts (Figures 4A,B). Specifically, LGG samples with a higher WHO grade, IDH wild type, 1p19q non-co-deletion had significantly higher risk scores than others, while there was no significant correlation between risk score and gender (Figures 4C–J). As a result, the risk score values were significantly related to the grade, IDH mutation, and 1p19q co-deletion status of LGG.

**TABLE 1:** Correlation between 14-GSRS genes risk scores and clinicopathological factors of glioma patients in the two cohorts.

	**Training set TCGA RNA-seq cohort (*n* = 447)**			**Validation set CGGA RNA-seq cohort (*n* = 513)**		
**Features**	**Low-risk score (*n* = 224)**	**High-risk score (*n* = 223)**	***P*-value**	**Low-risk score (*n* = 263)**	**High-risk score (*n* = 250)**	***P*-value**
**Age**			0.67			0.78
< = 45	132	135		190	187	
>45	92	88		73	63	
**Gender**			0.73			0.94
Female	99	99		108	109	
Male	125	124		155	141	
**WHO grade**			<0.001			<0.001
II	131	82		157	81	
III	93	141		106	149	
**IDH status**			<0.001			<0.001
Wild type	10	77		26	98	
Mutant	214	146		237	152	
**1p/19q status**			<0.001			<0.001
**Co-deletion**	138	11		129	33	
**Non-co-deletion**	86	212		134	217	

### Tumor-Infiltrating Immune Cells Profiles

**FIGURE 5:**
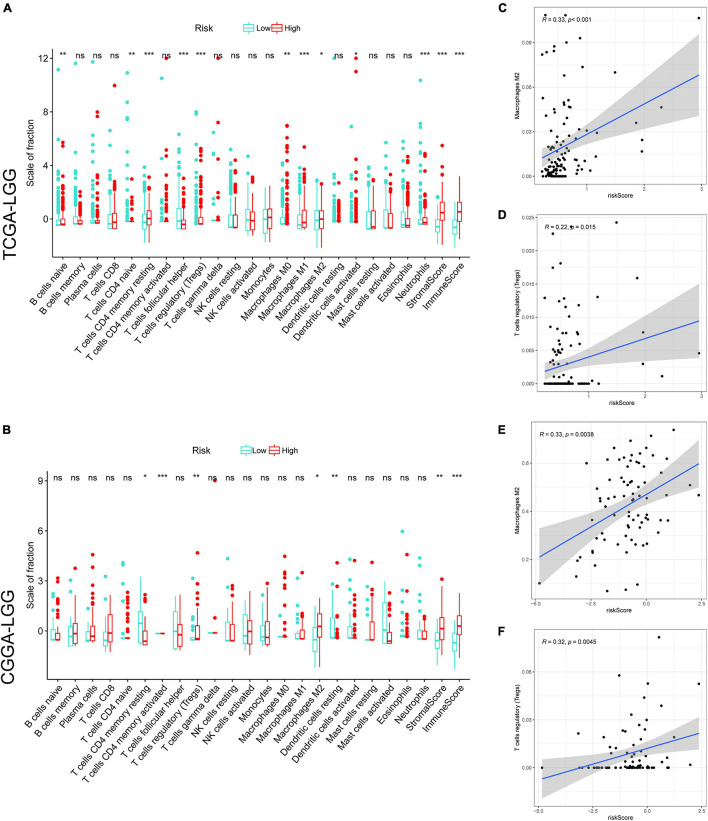
The correlation between tumor-infiltrating immune cells (TIICs) and GSRS. Difference analysis of 22 kinds of an abundance of TIICs, immune score, and stromal score in low and high-risk groups in training **(A)** and validation sets **(B)**. Spearman’s correlation analysis between risk score and M2 macrophages, regulatory T cells (Tregs) in TCGA **(C,D)** and CGGA cohorts **(E,F)**, each dot plot represents a subject, and the correlation is fitted into a straight blue line. *R*, rho; ***p < 0.001, **p < 0.01, *p < 0.05.

Tumor-infiltrating immune cells play an essential role in the TME. We investigated the relative fraction of 22 types of immune cells, calculated based on the “CIBERSORT” algorithm. Results of differential fraction analysis among low- and high-risk groups were shown in boxplots (Figures 5A,B); the abundances of M1, M2 macrophages, T cells regulatory (Tregs), memory resting CD4 T cells, and naive B cells in the high-risk group were significantly higher than that in the low-risk group. Furthermore, the stromal score captures the presence of stromal cells in tumor tissue, while the Immune score represents the infiltration of immune cells in the tumor area. We found that samples in high-risk groups had higher immune and stromal scores than samples in low-risk groups. It indicated that LGG samples with higher risk scores had higher infiltrating levels of stromal and immune cells. Moreover, as shown in the correlation analysis (Figures 5C–F), M2 macrophages and Tregs were confirmed to be significantly positively correlated with risk scores in TCGA and CGGA cohorts. These findings indicated that risk scores might affect the prognosis of LGG patients in association with increased M2 macrophages and Tregs.

**FIGURE 6:**
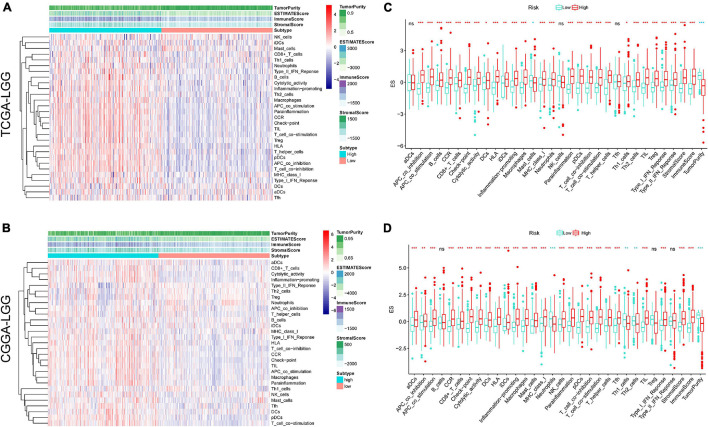
Single sample gene sets enrichment analysis (ssGSEA) of immune hallmarks. Heatmap of ssGSEA scores among low- and high-risk groups in training **(A)** and validation **(B)** sets (red = positive, blue = negative). Boxplot of ssGSEA scores, stromal score, immune score, and tumor purity among low- and high-risk groups in TCGA **(C)** and CGGA **(D)** cohorts. ***p < 0.001, **p < 0.01, *p < 0.05.

In addition, based on the ssGSEA scores, the activities and abundances of pathways, functions, or immunocytes were assessed quantitatively. Samples with higher ssGSEA scores indicated more infiltrating immune cells and activity of immune-related pathways. Samples in the high-risk group were associated with higher ssGSEA scores in terms of most immune cell types, as shown in the heatmaps (Figures 6A,B) and boxplots (Figures 6C,D). In general, LGG patients with higher risk scores tend to have a higher fraction of TIICs and more active immune-related pathways than other patients. The immunosuppressive TIICs (M2 macrophages and Tregs) exhibited significantly higher than baseline levels in the high-risk group.

### Association Between Risk Score and Immune Modulators

**FIGURE 7:**
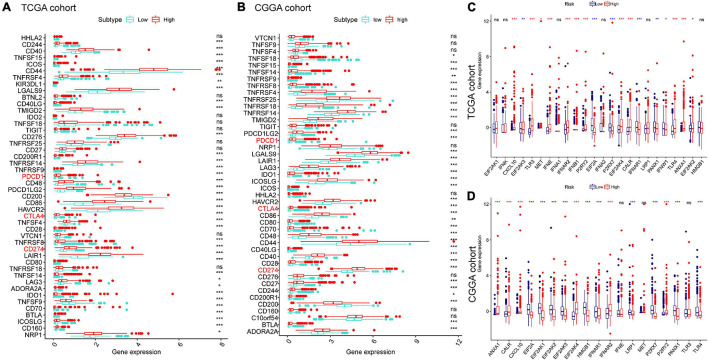
Association between risk subtypes and ICPs and ICD modulators. Differential expression of ICP genes among the risk subtypes in **(A)** TCGA and **(B)** CGGA cohorts. Differential expression of ICD modulator genes among the risk subtypes in **(C)** TCGA and **(D)** CGGA cohorts. *p < 0.01, **p < 0.001, and ***p < 0.0001.

We next explored their expression levels among different risk groups, considering the importance of ICPs and ICD modulators in anticancer immunity. Forty-seven ICPs-related genes were detected in the training and validation sets, of which 38 members in the TCGA and CGGA cohort (Figures 7A,B) were differentially expressed among the different risk groups. It is crucial that vital ICPs, such as PDCD1 (PD-1), CD274 (PD-L1), and CTLA4, were significantly upregulated in the high-risk group. Likewise, 18 and 16 ICD genes were expressed differently among two groups in TCGA and CGGA, respectively (Figures 7C,D). Therefore, the risk score value could reflect the expression levels of ICPs and ICD modulators and be treated as potential immune therapeutic biomarkers.

### The Association of Risk Score With Mutational Status

**FIGURE 8:**
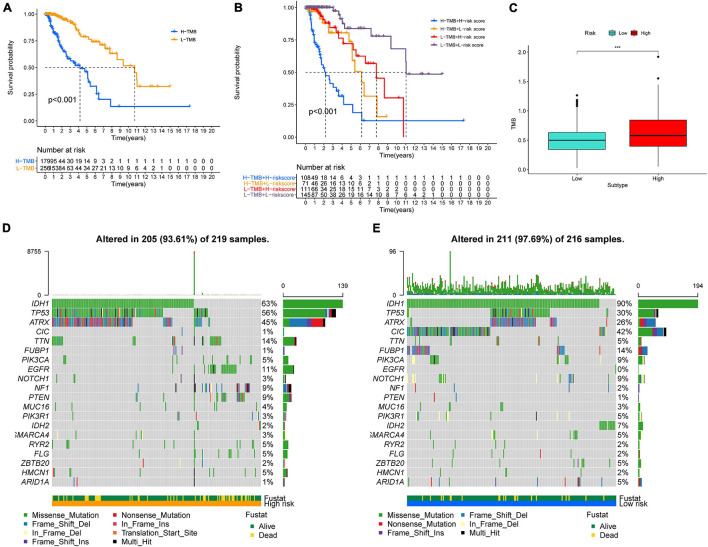
Association between risk subtypes and TMB and mutation. **(A)** Survival analysis of TMB and OS of the patients with LGG in TCGA. **(B)** K–M curves of TMB combining with risk score in TCGA-LGG. **(C)** Difference analysis of TMB among low- and high-risk subtypes in LGG patients. **(D)** Top 30 highly mutated genes in LGG low-risk group. **(E)** Top 30 highly mutated genes in LGG high-risk group. ***p < 0.0001.

Higher TMB and somatic mutation rates are associated with stronger antitumor immunity. First, we identified the prognostic value of TMB in LGG, and the survival analysis revealed that patients with higher TMB had a worse prognosis than samples with lower TMB in TCGA-LGG (Figure 8A). In addition, K–M curves of risk score combining with TMB indicated that patients with higher TMB and higher risk scores had worse OS than other subtypes, while the subtype of lower risk score with lower TMB was associated with the best prognosis (Figure 8B). Then, we explored the correlation between TMB and risk score in LGG. Difference analysis of TMB among low- and high-risk groups showed that TMB was significantly positively correlated with risk score (Figure 8C). Moreover, the mutation landscape was described in low- and high-risk groups. Twenty genes, including IDH1, TP53, and ATRX, were most frequently mutated in each subtype (Figures 8D,E). These findings suggested that the risk score based on GSRS can predict the TMB and somatic mutation rates in LGG patients and that patients with higher risk scores may respond positively to anticancer immunity.

### Gene Set Variation Analysis

**FIGURE 9:**
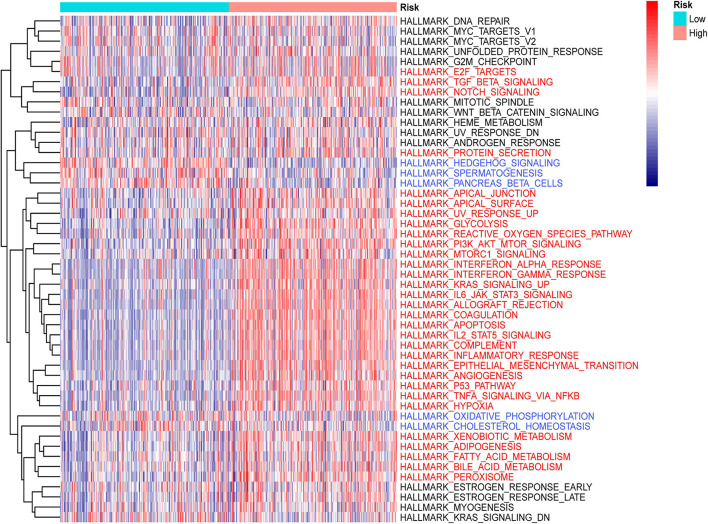
Heatmap for the contribution of gene set variation analysis (GSVA) scores of hallmarks in low- and high-risk groups. Terms with red color are significantly up-regulated in the high-risk group, blue color represent down-regulation in low-risk group.

Differences in pathway activities among low- and high-risk groups were scored using GSVA. We found that signaling pathways related to oncogenic transformation and tumorigenesis were primarily enriched in high-risk groups (Figure 9), such as the PI3k-Akt signaling pathway, P53 pathway, EMT pathway, and so on. These results suggested that risk score based on GSRS, as a novel biomarker for LGG, may be involved in some vital cancer-related signaling pathways.

### Identification of Hub Genes of Glutamatergic Synapse-Related Risk Signature

**FIGURE 10:**
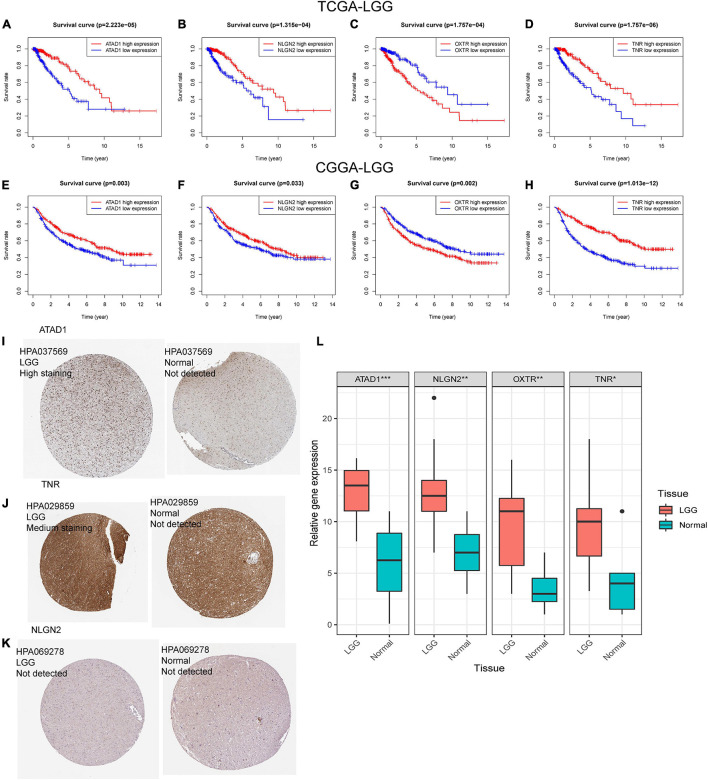
Verification of the prognostic value and expression of hub genes of GSRS. Survival analysis of ATAD1, NLGN2, OXTR, and TNR for patients in TCGA **(A–D)** and CGGA **(E–H)** cohorts. The protein expression level of ATAD1 **(I)**, NLGN2 **(K)**, and TNR **(J)** in normal and LGG tissues according to the HPA database. **(L)** The relative mRNA expression levels of ATAD1, NLGN2, OXTR, and TNR are compared among LGG and non-tumor tissues based on real-time PCR results. ***p < 0.001, **p < 0.01, *p < 0.05.

We then investigated the prognostic value of the 14-genes GSRS in the training and validation sets (Figures 10A–H). The K–M survival analysis of 14 genes (Table 2) indicated that the expression level of ATAD1, NLGN2, and TNR was positively correlated with the OS of patients with LGG, while patients with higher OXTR had inferior OS than others. As a result, ATAD1, NLGN2, TNR, and OXTR were identified as hub genes in GSRS. Subsequently, according to the HPA database, we explored the expression of the hub genes on protein level (Figures 10I–K), and proteins ATAD1 and TNR were upregulated in LGG tissues compared with normal brain tissues, while the expressions of protein NLGN2 in both LGG and normal tissues were not detected. However, there was no related data of OXTR in the HPA database.

**TABLE 2:** K–M survival analysis of 14 GSRS genes in TCGA and CGGA.

**Gene**	**TCGA**		**CGGA**	
	**HR (high)**	**Log-rank *P***	**HR (high)**	**Log-rank *P***
ADORA1	1.60	> 0.05	2.00	> 0.05
**ATAD1**	**0.49**	** < 0.001**	**0.52**	** < 0.01**
CCR2	1.60	> 0.05	1.80	> 0.05
DRD2	1.00	> 0.05	0.99	> 0.05
EGFR	1.30	> 0.05	1.22	> 0.05
GRIK2	0.48	> 0.05	0.55	> 0.05
GRIK3	1.20	> 0.05	1.44	> 0.05
KMO	1.62	> 0.05	1.11	> 0.05
**NLGN2**	**0.48**	** < 0.001**	**0.33**	** < 0.05**
NPY2R	1.61	> 0.05	1.88	< 0.001
**OXTR**	**2.30**	** < 0.001**	**2.90**	** < 0.01**
SHANK2	0.37	< 0.001	0.89	> 0.05
**TNR**	**0.42**	** < 0.001**	**0.36**	** < 0.001**
TSHZ3	1.50	< 0.05	1.22	> 0.05

*Factors with *p*-values less than 0.05 in TCGA and CGGA cohorts are marked in bold.*

Moreover, according to the real-time PCR results (Figure 10L), we detected the samples collected in our hospital; the control and primer sequences of four hub genes are as follows:

GAPDH: 5′-GGAGCGAGATCCCTCCAAAAT-3′ (For- ward), 5′-GGCTG TTGTCATACTTCTCATGG-3′ (Rev- erse).
ATAD1: 5′-ACACTGACCGATAAGTGGTATGG-3′ (For- ward), 5′-GTTGTAGCTTTATGGCAAGGGA-3′ (Rev- erse).
NLGN2: 5′-TGGTTCACCGACAACTTGGAG-3′ (For- ward), 5′-GCACGTAGAGGTTGAGGTACAG-3′ (Rev- erse).
OXTR: 5′-CTGCTACGGCCTTATCAGCTT-3′ (Forward), 5′-CGCTCCACATCTGCACGAA-3′ (Reverse).
TNR: 5′-AAGAATTGCTCGGAGCCCTAC-3′ (Forward), 5′-GCTGTACTCGCTGTCACAGAT-3′ (Reverse).

We found that mRNA expression of ATAD1, NLGN2, OXTR, and TNR were significantly upregulated in LGG compared with normal brain tissues, which were consistent with the expression patterns in the public database.

## Discussion

The formation of excitatory synapses between neurons and tumor cells promotes cancer growth (Li C. et al., 2020). Enhancing synaptogenesis and influencing synaptic transmission may participate in the growth and progress of gliomas (Miller, 2009). Moreover, synapses play a crucial role in many ways related to immune including self-tolerance, adaptive immunity, and prevention of autoimmunity (Siciliano et al., 2020). And the diversity and complexity of the immune context in TME influence metastasis and tumorigenesis of tumors (Siciliano et al., 2020). However, at present, though evidence has shown that glutamatergic synapses might affect the invasion and proliferation of glioma and have a significant correlation with the TIME (Venkataramani et al., 2019; Venkatesh et al., 2019), almost no specific biomarker had been developed based on GSRGs and immune status within gliomas. This study started with the gene sets associated with glutamatergic synapses to explore the expression, correlation, and genetic alterations of these genes in LGG. Then, we constructed a 14-genes GSRS and identified it as a novel prognostic biomarker in LGG by both internal and external validation.

Furthermore, we explored the association of risk scores with clinicopathological characteristics and immunity profiles. Moreover, the underlying molecular mechanism that might be regulated by GSRS was predicted by GSVA. In addition, the real-time PCR of tissues of the patients in our hospital confirmed the expression of hub genes in GSRS, and we also verified the expression of these genes at the protein level in LGG.

Most genes related to glutamatergic synapses were found differentially expressed among normal and tumor tissues. Furthermore, few CNV and single-nucleotide polymorphisms (SNP) of GSRGs in LGG suggested these genes may involve in the progression of glioma and indicated GSRGs maintained high genome stability that prevented the occurrence of genomic mutation. In this research, the 14-genes GSRS was constructed and verified. We found that the risk signature had a more vital prognosis prediction ability than other clinical independent prognostic factors, which might provide an effective individual mortality risk prediction and risk stratification in LGG patients. Consistently, the risk score was significantly increased with the WHO glioma grading, which indicated that risk signature can distinguish the degree of malignancy and may be involved in tumor progression. In addition, the status of IDH type and 1p/19q co-deletion among tumors could be distinguished by risk scores; IDH wild type and 1p19q non-co-deletion gliomas were the poor prognostic factors and had an inadequate response to traditional radiotherapy or chemotherapy of LGG patients (Li Y. et al., 2020). Consequently, LGG patients with higher risk scores may be less sensitive to radiotherapy or chemotherapy (Prados et al., 2009).

Tumor evolution to evade immune surveillance is a hallmark of carcinogenesis, and tumor-specific immunity can be directly affected by the modulation of the immune synapse between antigen-presenting cells and effector T cells (Lamantia et al., 2014). It had been reported that the induction of the immunosuppressive tumor microenvironment was owed mainly to the population of type M2 macrophages (Prados et al., 2009), and overcoming immunosuppressive tumor microenvironments is necessary for effective immunotherapy (Marigo et al., 2016). Similarly, the infiltration of M2 macrophages and Tregs was correlated with decreased tumor survival (Shah et al., 2011; Tian et al., 2020). In this study, high-risk patients tend to have higher M2 and Treg infiltration and a worse prognosis. At the same time, the risk score of LGG samples was positively correlated with the infiltrating level, including immune and stromal scores. It suggested a higher fraction of immune-inflammatory tumor-infiltrating cells that established immunosuppressive tumor microenvironment in high-risk groups. Therefore, repolarization of M2 into M1 macrophages and consuming the number and activity of infiltrating Tregs in the tumor could represent promising ways for patients with higher risk scores to treat LGG (Jézéquel et al., 2015).

On the flip side, ICB that aims to reverse signals from the immunosuppressive TME is being driven as a significant therapy (Huang et al., 2019), and ICD has been reported as a stimulant condition that changes “cold” immune microenvironment into a “hot” immune microenvironment (Duan et al., 2019). The expression of ICPs is vital for immune escape and treatment with ICB (Yang et al., 2021). Therefore, the focus of recent developments in immunotherapy for cancer had shifted toward immune checkpoint inhibitors (ICIs; Wierstra et al., 2019). Based on our constructed GSRS in this research, the expression of vital ICPs (PD-1, PD-L1, and CTLA4) and TMB correlated significantly with a risk score, and the results indicated that high-risk patients tend to be in a state of more sensitivity to ICB therapy. The expression level of ICD had no significant difference among low- and high-risk groups, which may not provide sufficient theoretical support for further research. The potential complex interaction of GSRS developed in this study with ICPs and immune infiltration might be a promising research direction to improve the effect of ICB for solid cancers.

When investigating potential mechanisms related to GSRS, we found that the highly enriched terms in high-risk samples were mostly cancer-related pathways. Recent studies have shown that the formation of synaptic potassium current contributes to the transmission of neuron-glioma synapse (NGS) signal, and calcium ion, as a signal communication between glioma cells, plays a crucial role in activating the whole glioma network (Venkataramani et al., 2019; Venkatesh et al., 2019), given the existence of glutamatergic synapse among TME of gliomas and the effect of potassium and calcium on the electrochemical signal transduction in glioma. Hamerlik et al. (2012) mentioned that glutamate might affect the proliferation, invasion, and angiogenesis of glioblastoma by activating the epidermal growth factor receptor (EGFR) signaling pathway.

Due to the highly heterogeneous character of gliomas (Hamerlik et al., 2012), it is not reliable to use a single differentially expressed gene as the biomarker in individual glioma patients. Simultaneously, it is difficult to discriminate the subtypes in glioma using classical biotyping methods and molecular schedules (Radoul et al., 2021). In this study, a novel prognostic biomarker constructed in LGG could distinguish the malignant degree and immune status of glioma and predict the effect of ICB therapy for patients.

However, it cannot be neglected that the current research on glioma-related electrophysiology is in the pioneering and developing stage, which needs further multicenter, prospective, and well-designed studies.

## Conclusion

Based on 14 GSRGs, a prognostic signature was constructed and validated for the sake of predicting the OS for patients with LGG. In addition, combining immune profiles with genetic multi-omics assays, our GSRS provided a novel and comprehensive perspective for clarifying the potential mechanisms underlying the prognosis of LGG.

## Data Availability Statement

The original contributions presented in the study are included in the article/Supplementary Material, further inquiries can be directed to the corresponding authors.

## Ethics Statement

Written informed consent was obtained from the individual(s) for the publication of any potentially identifiable images or data included in this article.

## Author Contributions

LY, YX, QC, and DT contributed to the conception and design of the study. LW, JY, PH, YW, CZ, and FY contributed to the analysis and interpretation of data. All authors read and approved the final manuscript.

## Conflict of Interest

The authors declare that the research was conducted in the absence of any commercial or financial relationships that could be construed as a potential conflict of interest.

## Publisher’s Note

All claims expressed in this article are solely those of the authors and do not necessarily represent those of their affiliated organizations, or those of the publisher, the editors and the reviewers. Any product that may be evaluated in this article, or claim that may be made by its manufacturer, is not guaranteed or endorsed by the publisher.

## Footnotes

1
http://cancergenome.nih.gov/

2
http://www.cgga.org.cn

3
http://www.broad.mit.edu/gsea/msigdb/

4
http://www.string-db.org/

5
http://www.cbioportal.org/

6
https://www.proteinatlas.org/

## Supplementary Material

Supplementary Figure 1 CNV and SNP of GSRGs in LGG according to cBioPortal database. T, tumor; N, normal; CNV, copy number variation; SNP, single-nucleotide polymorphism.

## Abbreviations

LGG: lower-grade glioma
WHO: World Health Organization
TIME: tumor immune microenvironment
TME: tumor microenvironment
TCGA: The Cancer Genome Atlas
CGGA: the Chinese Glioma Genome Atlas
GTEx: Genotype-Tissue Expression
GSRGs: glutamatergic synapse-related genes
GSRS: glutamatergic synapse-related risk signature
OS: overall survival
TIICs: tumor-infiltrating immune cells
ssGSEA: single-sample gene-set enrichment analysis
GSVA: gene set variation analysis
ICPs: immune checkpoints
ICD: immunogenic cell death
GO: Gene Ontology
KEGG: Kyoto Encyclopedia of Genes and Genomes
ICIs: immune checkpoint inhibitors.
