# Supplementary figures and images for: Development and Verification of Glutamatergic Synapse-Associated Prognosis Signature for Lower-Grade Gliomas
Source: Front Mol Neurosci. 2021 Oct 28;14:720899. doi: 10.3389/fnmol.2021.720899 (PMC8581158; doi:10.3389/fnmol.2021.720899)

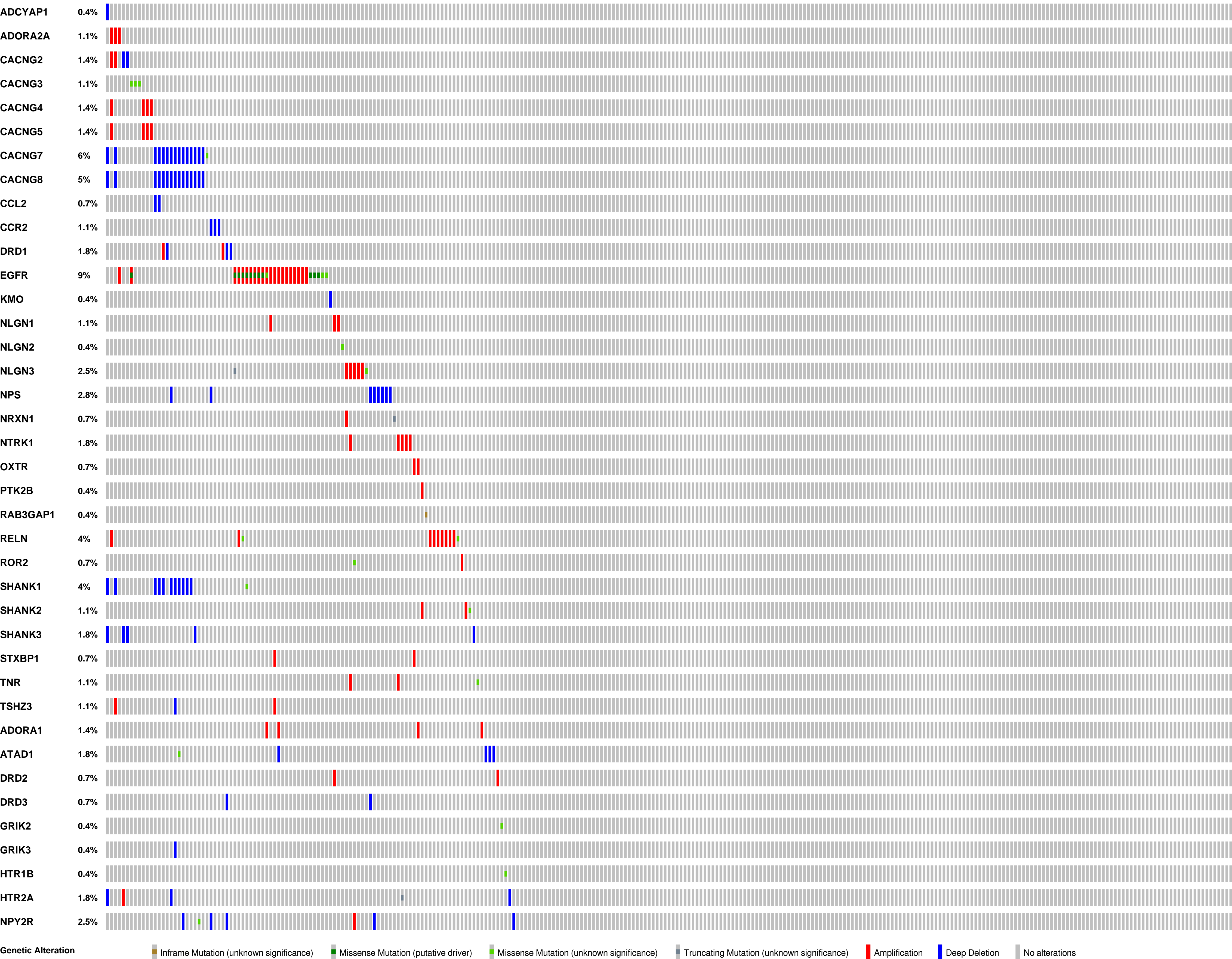

Supplement: Supplementary Figure 1 — CNV and SNP of GSRGs in LGG according to cBioPortal database. T, tumor; N, normal; CNV, copy number variation; SNP, single-nucleotide polymorphism. [file Image_1.PDF]
