# Supplementary material for: Development and Verification of Glutamatergic Synapse-Associated Prognosis Signature for Lower-Grade Gliomas
Source: Front Mol Neurosci. 2021 Oct 28;14:720899. doi: 10.3389/fnmol.2021.720899 (PMC8581158; doi:10.3389/fnmol.2021.720899)
Supplement: Supplementary file 2 [file Table_1.docx]

**Table S1: The expression regulation of 39 glutamatergic synapse-related genes (GSRGs).**

| GSRGs | Up- or Down-regualted in LGG | logFC |
| --- | --- | --- |

| DRD2 | Down | -3.436591982 |
| --- | --- | --- |
| DRD3 | Down | -2.739318257 |
| ADORA2A | Down | -7.681283953 |
| RELN | Down | -3.488812291 |
| DRD1 | Down | -2.467234777 |
| PTK2B | Down | -2.056658566 |
| ADCYAP1 | Down | -1.967179532 |
| SHANK1 | Down | -1.618194315 |
| SHANK3 | Down | -1.45902954 |
| NTRK1 | Down | -1.453965157 |
| STXBP1 | Down | -1.409778946 |
| HTR1B | Not significant | -0.931275534 |
| GRIK2 | Not significant | -0.395265085 |
| HTR2A | Not significant | 0.332552799 |
| KMO | Not significant | -0.929695242 |
| CACNG3 | Not significant | -0.842652043 |
| SHANK2 | Not significant | -0.6581789 |
| CACNG8 | Not significant | -0.244287439 |
| RAB3GAP1 | Not significant | 0.534254062 |
| CACNG2 | Not significant | 0.708612057 |
| CCL2 | Not significant | 0.951234655 |
| ATAD1 | Up | 1.034707943 |
| GRIK3 | Up | 3.175742424 |
| CACNG7 | Up | 1.166612197 |
| OXTR | Up | 1.303992632 |
| NLGN2 | Up | 1.366050597 |
| TSHZ3 | Up | 1.697424376 |
| NLGN1 | Up | 1.827999852 |
| NLGN3 | Up | 2.547535914 |
| CACNG4 | Up | 2.836268794 |
| TNR | Up | 3.635273062 |
| EGFR | Up | 4.984790725 |
