# Supplementary material for: Development and Verification of Glutamatergic Synapse-Associated Prognosis Signature for Lower-Grade Gliomas
Source: Front Mol Neurosci. 2021 Oct 28;14:720899. doi: 10.3389/fnmol.2021.720899 (PMC8581158; doi:10.3389/fnmol.2021.720899)
Supplement: Supplementary file 3 [file Table_2.docx]

Table S2. Genes and corresponding coefficients in glutamatergic synapse-related risk signature (GSRS).

| Gene | Coef |
| --- | --- |
| EGFR | 0.001905002 |
| CCR2 | 0.393583795 |
| ATAD1 | -0.034349733 |
| NLGN2 | -0.011870027 |
| SHANK2 | -0.141170876 |

| OXTR | 0.02447963 |
| --- | --- |

| GRIK2 | -0.022043335 |
| --- | --- |

| TNR | -0.000741699 |
| --- | --- |

| KMO | 0.632651505 |
| --- | --- |

| DRD2 | 0.163416365 |
| --- | --- |

| NPY2R | 0.024335421 |
| --- | --- |

| ADORA1 | 0.000448907 |
| --- | --- |

| GRIK3 | 0.01284423 |
| --- | --- |

| TSHZ3 | 0.021074875 |
| --- | --- |
